# Disconnections in personal neglect

**DOI:** 10.1007/s00429-022-02511-z

**Published:** 2022-06-07

**Authors:** S. Bertagnoli, V. Pacella, E. Rossato, P. M. Jenkinson, A. Fotopoulou, M. Scandola, Valentina Moro

**Affiliations:** 1 NPSY-Lab.VR, Department of Human Sciences, University of Verona, Lungadige Porta Vittoria, 17, 37129 Verona, Italy; 2 Groupe d’Imagerie Neurofonctionnelle, Institut des Maladies Neurodégénératives-UMR 5293, CNRS, CEA University of Bordeaux, CS 33076 Bordeaux, France; 3 Department of Rehabilitation, IRCSS Sacro Cuore Don Calabria, 37024 Negrar, Verona Italy; 4 Institute for Social Neuroscience, Ivanhoe, Melbourne, VIC Australia; 5 Department of Clinical, Educational and Health Psychology, University College of London, London, WC1E 6BT UK

**Keywords:** Personal neglect, White matter disconnection, Multivariate voxel lesion symptom mapping, Heschl’s gyrus, Thalamus, Fornix

## Abstract

Personal neglect is a disorder in the perception and representation of the body that causes the patients to behave as if the contralesional side of their body does not exist. This clinical condition has not been adequately investigated in the past as it has been considered a symptom of unilateral spatial neglect, which has mainly been studied with reference to extrapersonal space. Only a few studies with small samples have investigated the neuroanatomical correlates of personal neglect, and these have mainly focused on discrete cortical lesions and modular accounts, as well as being based on the hypothesis that this disorder is associated with somatosensory and spatial deficits. In the present study, we tested the novel hypothesis that personal neglect may be associated not only with discrete cortical and subcortical lesions, but also with disconnections of white matter tracts. We performed an advanced lesion analyses in a large sample of 104 right hemisphere damaged patients, 72 of whom were suffering from personal neglect. Results from the analyses of the grey and white matter were controlled for co-occurrent clinical variables such as extrapersonal neglect, anosognosia for hemiplegia and motor deficits, along with other lesion-related variables such as lesion size and the interval from the lesion onset to neuroimaging recordings. Our results reveal that personal neglect is associated with lesions in a medial network which involves the temporal cortex (Heschl’s gyrus), the ventro-lateral nuclei of the thalamus and the fornix. This suggests that personal neglect involves a convergence between sensorimotor processes, spatial representation and the processing of self-referred information (episodic memory).

**Supplementary Information:**

The online version contains supplementary material available at 10.1007/s00429-022-02511-z.

## Introduction

One of the most fascinating phenomena of the human mind is the cognitive process by which an individual’s experiences of themselves and their body are integrated (Orfei et al. 2007). Neurological damage can lead to anomalies in the perception and representation of one's own body, such as in the case of personal neglect (PN). Patients suffering from PN behave as if the contralesional part of their body does not exist. For example, they might appear with only half of their face shaved or made up, their hair combed only on the ipsilesional side or with their glasses misplaced on the contralesional side of their head. They might only wear a slipper or an earring on the ipsilesional side. Even their posture may suggest indifference relating to the contralesional side of their body, as both standing and sitting, they tend to place themselves in the so-called “three-quarter” position, with the ipsilesional part of the body leaning forward and the contralesional part left behind. This frequently results in accidents and traumas which are due to the patient neglecting the position of their body, and it may also negatively impact motor recovery (Committeri et al. 2018).

In the first reports of patients displaying PN, their failure to explore the contralesional parts of the body was described (Zingerle 1913; Cutting 1978). Since then, subsequent definitions have maintained this aspect as the main focus of the condition (e.g.Beschin and Robertson 1997; McIntosh et al. 2000; Committeri et al. 2007; Caggiano et al. 2014), although various other, specific aspects have been referred to, such as patients’ inability to recognise and use their paretic limbs (in the absence of severe motor deficits, Guariglia and Antonucci 1992; Iosa et al. 2016; Cocchini and Beschin 2022), or to orient their attention towards the contralesional side of their body (Marangolo et al. 2003; de Vignemont 2010; Ronchi et al. 2018) or mentally represent only this part of their body (Bisiach and Luzzati 1978; Baas et al. 2011; Reinhart et al. 2012; Di Vita et al. 2017).

PN has been also described in terms of hemisomatoagnosia (or asomatognosia, Vallar 1998; Vallar and Calzolari 2018), namely, as a lack of awareness of the contralesional body part. Nevertheless, several experimental studies have shown the existence of dissociations between the two clinical conditions (Moro et al. 2004; Spinazzola et al. 2020), although they are both typically present as consequences of strokes in the right hemisphere. For example, patients with personal neglect (but without asomatognosia) recognise their contralateral limb when it is moved so that they can see it in the ipsilesional space (Moro et al., 2004). Conversely, patients suffering from asomatognosia do not recognise the arm as their own (Jenkinson et al. 2018) even when their attention is focused on that body part. Specifically, asomatognosia refers to a disturbance relating to the feeling of ownership of the affected body part, with patients reporting seeing it fading or disappearing or missing completely. These symptoms are not present in PN.

Dissociations have also been reported in cases of extrapersonal neglect, when there is a consistent reduction in the processing of information coming from the contralesional side of external space in comparison with the information coming from the ipsilesional side (Cubelli 2017). Dissociations have been found both during clinical assessments (Guariglia and Antonucci 1992; Baas et al. 2011; Di Vita et al. 2017) and in studies of lesional correlates (Committeri et al. 2018). In a recent review of the literature on the topic (Caggiano and Jehkonen 2018), it appears that PN is less frequently diagnosed than extrapersonal neglect, although a frequency of 30.8% is reported in patients with right hemispheric lesions. A lack of adequate tests to evaluate the condition has probably also contributed to the syndrome being underestimated in the past (Guariglia and Antonucci 1992; Committeri et al. 2018). In addition, PN is often associated with other deficits (such as motor, somatosensory and visual field deficits, extrapersonal neglect and anosognosia for hemiplegia) and these may make the symptoms difficult to isolate.

The hypothesis at the basis of the present study is that PN is a disconnection syndrome which is not associated with discrete grey matter lesions, but instead involves a right hemisphere network of cortical and subcortical structures contributing to body representation. Related disconnection hypotheses have been recently supported in other syndromes, such as spatial neglect (Thiebaut de Schotten et al. 2005), anosognosia for hemiplegia (Pacella et al. 2019; Monai et al. 2020) and disorders in the sensations of body ownership (Moro et al. 2022). Previous neuroanatomical data on PN correlates also support this hypothesis, since they suggest a role played by multimodal areas (i.e. temporo-parietal junction, Baas et al. 2011; supramarginal gyrus, Committeri et al. 2007) and the underlying white matter connections (Baas et al. 2011; Committeri et al. 2007) in the syndrome, along with lesions in the somatosensory cortex and superior temporal sulcus (Azouvi et al. 2002; Rousseaux et al. 2015). Based on this evidence, recent revisions of the literature have suggested that PN is due to both hodological (i.e. caused by white matter disconnection) and topological (i.e. caused by grey matter lesion) mechanisms (Caggiano and Jehkonen 2018; Committeri et al. 2018).

However, certain limitations persist in previous results, for several reasons. Firstly, to date neuroanatomical studies on PN have been conducted on small samples, with a maximum of 30 patients presenting with the symptoms (Azouvi et al. 2002; Buxbaum et al. 2004; Committeri et al. 2007; Baas et al. 2011; Rousseaux et al. 2015; Caggiano et al. 2020); Furthermore, the results of these studies focused on the identification of discreet cortical lesions and did not analyse white matter disconnections in a specific way. In this study, a disconnection approach was used that allows us to go beyond the descriptions of the sites of lesions and analyse the actual probability of disconnection of specific white matter tracts. Finally, in previous studies, the co-occurrent neuropsychological symptoms were usually considered in clinical comparisons between groups of patients with and without PN symptoms, while in this study these were directly entered into neuroanatomical analyses as covariates, or alternative models of causation.

The present study aimed thus to overcome the limitations of previous studies by investigating the neural correlates of PN in a sample of 104 right hemisphere damaged patients, 72 of whom showed a pathological score in a validated, neuropsychological test assessing PN (Comb subtest of the Comb and Razor test, McIntosh et al. 2000). A multivariate voxel lesion symptoms mapping approach (LESYMAP) with sparse canonical correlations (Mirman et al. 2018) was used to identify the grey matter structures whose lesions correlate with PN symptoms in the whole group. The multivariate approach is considered to be statistically and conceptually more adequate than the univariate counterparts for lesion analyses. Indeed, while the univariate voxel lesion symptom mapping techniques assume independency among voxels, the multivariate voxel lesion symptom mapping approach detects which group of voxels together contribute to the emergence of behavioural deficits, assuming a statistical dependency (Pustina et al. 2018). This approach allows us to consider that lesions usually extend to more than a voxel and, consequently, the probability that a voxel is lesioned is dependent on the probability of the surrounding voxels’ conditions. For the white matter, the Tractotron software (Foulon et al. 2018) was used to identify the probability of disconnections for each known tract and each patient, taking into account the contribution of clinical and neuropsychological variables. Finally, a series of linear models were performed, and comparisons were made to identify which of the structures resulting from the anatomical analyses explain PN more clearly than the clinical symptoms, and to ascertain whether these are integrated into a network.

## Materials and methods

### Design and statistical analysis

The aim of the study was to explore the grey matter lesions and white matter disconnections involved in personal neglect and to investigate whether the damaged structures are integrated into a network. For this purpose, we collected neuroimaging and clinical data from a large sample of right hemisphere stroke patients and analysed their lesion and disconnection maps in correlation with their scores on the Comb subtest of the Comb and Razor test (see McIntosh et al. 2000, and below).

A multivariate voxel lesion symptom mapping approach (LESYMAP ver. 0.0.0.9221 with sparse canonical correlations; Mirman et al. 2018) was carried out on the scores of the Comb subtest (McIntosh et al. 2000) as continuous predictors (Bates et al. 2003; Rorden et al. 2007) of grey matter lesions. We also recorded the lesion size and took into account the number of voxels of each lesion as a nuisance variable.

The white matter tracts associated with PN were extracted for each known tract and for each patient via Tractotron software (Foulon et al. 2018), giving the probability of a disconnection. Then, grey matter structures and white matter tracts were analysed by means of linear models to identify the structures which may account for PN while taking into account any nuisance clinical variables. Finally, the presence of an integration of these structures in a network was analysed by means of a comparison of the models. All statistical analyses, unless otherwise specified, were computed on R version 3.6.3 (R Core Team 2020).

### Patients

Data from 104 stroke patients with unilateral right hemisphere damage were collected as part of a joint project involving two centres based in Italy and the UK over a period of 10 years. The inclusion criteria were: (1) unilateral right hemisphere damage, secondary to a first-ever stroke, as confirmed by clinical neuroimaging and (2) right hand dominance. The exclusion criteria were: (1) a previous history of neurological or psychiatric illness; (2) medication causing severe cognitive or mood side-effects; (3) severe language impairment, general cognitive impairment, or mood disturbances that would potentially preclude the completion of the assessments made during the study and (4) left hand dominance.

The clinical MRI or CT neuroimaging data were available for all of the patients. Their clinical and anatomical data had been described in two previous studies which focused on the neural correlates and diagnosis of anosognosia for hemiplegia (Pacella et al. 2019; Moro et al. 2021) and disorders in the sense of body ownership (Moro et al. 2022) and the data are shown in Table 1. As the data were collected from two different stroke recovery units, we took into account the neurological and neuropsychological tests that were most commonly administered to all of the patients in the centres and investigated the symptoms which are more frequently associated with PN (i.e. extrapersonal neglect, anosognosia for hemiplegia, sense of ownership and motor deficits). All of the patients gave written informed consent and the research was conducted in accordance with the guidelines of the Declaration of Helsinki (2013) and approved by the Local Ethical Committees of each centre.

**Table 1:** Data from the clinical and neuropsychological assessments

	104 patients	72 PN+	32 PN−
	Mean (Std. dev)	Mean (Std. dev)	Mean (Std. dev)
Demographic and clinical			
Age (years)	66.42 (± 13.98)	66.21 (± 13.95)	66.91 (± 14.25)
Education (years)	10.96 (± 3.69)	10.91 (± 3.63)	10.99 (± 3.99)
Interval (days)	44.72 (± 80.33)	47.64 (± 91.99)	38.16 (± 44.68)
Lesion size (voxels)	124,754.39 (± 121,430.57)	123,858.99 (± 122,206.72)	126,769.06 (± 121,580.79)
Personal neglect			
Comb subtest	− 0.25 (± 0.35)	− 0.42 (± 0.24)	0.13 (± 0.23)
One Item test (range 0–3)	0.41 (± 0.73)	0.51 (± 0.82)	0.19 (± 0.4)
Extrapersonal neglect			
Line bisection test (max = 9)	2.94 (± 2.99)	2.58 (± 2.75)	3.75 (± 3.4)
Line crossing (max = 36)	23.29 (± 11.54)	23.25 (± 10.99)	23.38 (± 12.87)
Anosognosia			
Bisiach for AHP score (max = 3)	1.11 (± 1.28)	1.22 (± 1.3)	0.86 (± 1.2)
DSO (range 0–6)	1.62 (± 1.43)	1.69 (± 1.39)	1.47 (± 1.52)
Motor index (LUL)			
MRC (range 0–5)	0.41 (± 0.76)	0.36 (± 0.72)	0.53 (± 0.84)

Mean and standard deviation are reported for each variable. Data from the whole sample are reported in the second column. In the third and fourth column, there are data from patients with (PN +) and without (PN−) personal neglect, respectively. PN = personal neglect; Std. dev. = standard deviation; AHP = anosognosia for hemiplegia; DSO = disturbance relating to the feeling of ownership of the affected body part; LUL = left upper limb; MRC = Medical Research Council Motricity scale. In the PN + group, 44 patients fail in both the line bisection and line crossing tests (Wilson et al. 1987), 21 only in line bisection, 3 only in line crossing and 5 do not suffer from extrapersonal neglect. In the PN− group, 15 patients fail in both the tests, 7 only in line bisection, 2 only in line crossing and 8 do not show extrapersonal neglect

### Neurological and neuropsychological assessment

The patients were assessed for PN by means of two tasks (Table 1): the Comb subtest of the Comb and Razor/Compact Test (McIntosh et al 2000, data from the Razor/Compact subtest were not available for 49 out of 104 patients) and the One Item test (Bisiach et al. 1986a, b).

In the Comb test, patients are asked to comb their hair for 30 s without stopping. The examiner records all the contacts that the comb makes in terms of whether they are on the left or right side of the head or in the centre (i.e. ambiguous contact). Although the original version of the task (Beschin and Robertson 1997) suggests the use of two subtests (i.e. Comb and Razor/Compact), the two subtests are highly correlated (McIntosh et al. 2000; Beschin and Robertson 1997) and several previous studies have used only one subtest (e.g. Moro et al. 2022; Moro and Besharati 2021; Pacella et al. 2019; D’Imperio et al. 2017a, b; Caggiano et al. 2020). The choice of the Comb subtest guarantees the possibility of using the same task for both males and females and to exclude any effects of sensory deficits (as the upper part of the face has bilateral innervation).

The score is calculated by means of an algorithm which identifies the proportion of contacts on the left and on the right side of the head with respect to the total score (left contacts − right contacts/left + ambiguous + right contacts). In this way, the score can range from − 1 (i.e. the maximum score indicating a left side bias) to + 1 (the maximum score indicating a right side bias; McIntosh et al. 2000). In the present study, this value was then converted in *z*-scores and the sign was inverted to have all of the positive scores showing a greater degree of personal neglect than the mean of the sample, and the negative scores showing less personal neglect than the mean of the sample. This allowed us to overcome the limits associated with the use of a cutoff to distinguish the patients into two groups, a procedure that does not take into consideration the scores immediately above or below the cutoff limits, and thus with performance that may be very similar. Continuous analyses were carried out on the whole group.

To verify the diagnosis of PN (see “Discussion” for this point), also the One Item test (Bisiach et al. 1986a) was administered. In this task, the patient is asked to touch their left hand with their right hand and the action is scored as follows: 0 = if the target is promptly reached without hesitation; 1 = the target is reached with some hesitation and searching; 2 = the search is interrupted before the target is reached and 3 = no movement towards the target is performed.

As measures of extrapersonal neglect, we used each patient’s score on the line crossing and line bisection subtests of the BIT (Behavioral Inattention Test, Wilson et al. 1987). Plegia of the contralesional upper limb was assessed by means of the Medical Research Council 5-point scale (MRC, Matthews 1976), ranging from 5 (normal functioning) to 0 (no movement). Anosognosia was assessed by means of the Bisiach interview for anosognosia for hemiplegia (AHP). Patients were required to verbally answer a 4-point interview about their current condition: a score of ‘0’ indicated a spared consciousness of the disease (= the disorder is spontaneously reported or mentioned by the patient following a general question about his/her condition), a score of ‘1’ was assigned when patients referred to their disability only after specific questions about the strength of their left limbs, while patients scoring ‘2’ or ‘3’ were considered anosognosic since their awareness of the disease only emerged after a demonstration using a routine technique involving a neurological examination (score 2) or not emerging at all (score 3, Bisiach et al.1986b).To assess the sense of body ownership, three questions were asked with reference to the patient’s left hand (moved to the ipsilesional field to reduce neglect): (1) “Is this your hand?”; (2) “Do you ever feel as if this was not your hand?” and (3) “Does it belong to someone else?”. The patients’ responses to each question were scored by two expert clinicians, with 0 = the patient recognises the arm as belonging to him/her; 1 = uncertain answers indicating doubts about ownership and 2 = responses indicating disownership and/or attribution of the arm to somebody else (min score = 0; max score = 6).

Missing data (education: 10.58% of the whole group; lesion-assessment interval: 3.85%; MRC 4.81%; One Item test: 17.31%; line crossing: 0.96%; line bisection: 3.85%; Bisiach test for AHP: 3.85%, and disorders in the sense of body ownership: 13.46%) were imputed by means of multivariate imputation by chained equations (van Buuren and Groothuis-Oudshoorn 2011), after having checked if they were missing at random (Rubin 1987, 1996) by testing for associations between missing and observed data. This methodology is modelled on a variable-by-variable basis by a set of conditional densities, one for each incomplete variable. Starting from an initial imputation, this method infers imputations by iterating over the conditional densities (Rubin 1987, 1996).

### Lesion delineation

The patients’ neuroimaging data were acquired via computerised tomography (CT, n.97) and magnetic resonance (MRI, n.7) and lesions were segmented and co-registered using the manual procedure already described by Moro and colleagues (Moro et al. 2016). The lesion drawing was performed blindly and independently by two of the authors (VM, SB). In cases of disagreement on a lesion drawing, a third anatomist’s opinion was consulted (< 10%) and the differing opinions were discussed until an agreement was reached.

Scans were registered on the ICBM152 template of the Montreal Neurological Institute, furnished with the MRIcron software (ch2, http://www.mccauslandcenter.sc.edu/mricro/mricron/). First, the standard template was rotated on the three planes (size: 181 × 217 × 181 mm, voxel resolution: 1 mm^2^) to match the orientation of patient’s MRI or CT scan. The lesions were outlined on the axial slices of the rotated template. The resulting volumes were then rotated back into the canonical orientation, in order to align the volumes of the lesions of each patient to the same stereotaxic space. Finally, to remove the voxels of lesions outside the white and grey matter brain tissue, the volumes were filtered by means of custom masks based on the ICBM152 template.

### Lesion symptom mapping

Only the voxels that were lesioned in at least 10% of the 104 patients were taken into account (Mirman et al. 2018).

The multivariate voxel lesion symptom mapping procedure uses sparse canonical matrices as an optimisation routine based on machine learning principles and a fourfold cross-validation technique (for details, see Mirman et al. 2018). The outcomes of these analyses were superimposed onto T1 templates and then overlapped onto the Automatic Anatomical Labeling (AAL) template (Tzourio-Mazoyer et al. 2002) to provide the proportion [0–1] of the lesion for each patient and the lesioned grey matter area.

### White matter disconnection

We used the “Tractotron” tool of the BCBToolkit (Foulon et al. 2018, http://www.toolkit.bcblab.com) to ascertain the severity of white matter disconnections in all of the 104 patients. Based on a comprehensive white matter atlas (Rojkova et al. 2016), Tractotron provides the probability of disconnection for every known white matter tract in a 0–1 range. The probability corresponds to each patient’s lesioned voxel with the highest percentage value. Only the tracts disconnected in at least 20% of the patients were included in the following analysis.

### Statistical analyses

Statistical analyses were carried out in two steps. First, to explore which tracts and structures significantly contribute to PN, each of the 34 tracts and 5 grey matter structures found in the previous steps were used in linear regression models as the independent variable, along with each patient’s lesion size, interval, and their scores at the MRC, Bisiach test for AHP and line bisection as the independent control variables. The choice of using the line bisection test as a measure of spatial bias is based on recommendations by Sperber and colleagues (2020). Multivariate voxel lesion symptom mapping analysis should not correct for neuropsychological assessments whose neural correlates might be involved with the target performance, since doing so might introduce biases and artefacts that invalidate the analysis (Sperber et al. 2020). Therefore, because the above-mentioned literature suggests that PN is associated with the temporo-parietal junction (Baas et al. 2011; Committeri et al. 2007), we decided to use the line bisection task (which is associated with dorsolateral prefrontal and parietal areas, Molenberghs et al. 2012; Pedrazzini and Ptack 2020) rather than the line crossing task, whose neural correlates are very close to those of PN (infero-parietal areas, Verdon et al. 2010).

In this analysis, all the clinical scores were converted into *z*-scores to avoid potential statistical biases caused by differing ranges and means. The *z*-scores of the Comb subtest were used as the dependent variable for each regression.

After this, as a second step, all the structures and tracts whose lesion or disconnection was significantly connected with the Comb subtest performance were considered as independent variables in a model along with the clinical variables as control independent variables (*z*-scores of the lesion size, interval, motricity, Bisiach test for AHP and line bisection) and the Comb subtest as dependent variable. This model was compared to a model taking into consideration only the clinical variables (without lesions) and the Comb subtest as dependent variable. This allowed us to understand whether the brain network associated with PN explained the symptoms more clearly beyond the co-occurrent clinical symptoms.

## Results

To check whether data imputation had introduced biases in the behavioural data, the distribution was compared to the original data distribution with missing data by means of Kolmogorov–Smirnov statistical tests. These are non-parametric tests which make it possible to check whether two distributions come from two different statistical populations (alternative hypothesis). In all cases, the comparison between the original and the imputed data was not statistically significant (all *p* = 1, all *D* < 0.03), suggesting that the data imputation did not introduce biases.

Continuous multivariate analysis with LESYMAP showed the correlations between the lesions on the grey matter structures and the behavioural data obtained from the Comb subtest. The cortical areas emerging from the analysis were the superior temporal gyrus (voxels > 0 = 23, %voxels > 0 = 0.1%), the Heschl’s gyrus (voxels > 0 = 353, %voxels > 0 = 18.2%), the Rolandic operculum (voxels > 0 = 142, %voxels > 0 = 1.3%), the hippocampus (voxels > 0 = 20, %voxels > 0 = 2.6%), and the insula (voxels > 0 = 235, %voxels > 0 = 1.7%). Subcortical structures such as the pallidum (voxels > 0 = 24, %voxels > 0 = 1.1%) and thalamus (voxels > 0 = 1117, %voxels > 0 = 13.3%) were also involved in the symptom (Table SM1 in the Supplementary Materials for details).

For the white matter, Tractotron shows 34 different tracts that might be involved in PN and disconnected in at least 20% of the patients. The probability of disconnection is shown in Table SM2 in the Supplementary Materials.

**Fig. 1:**
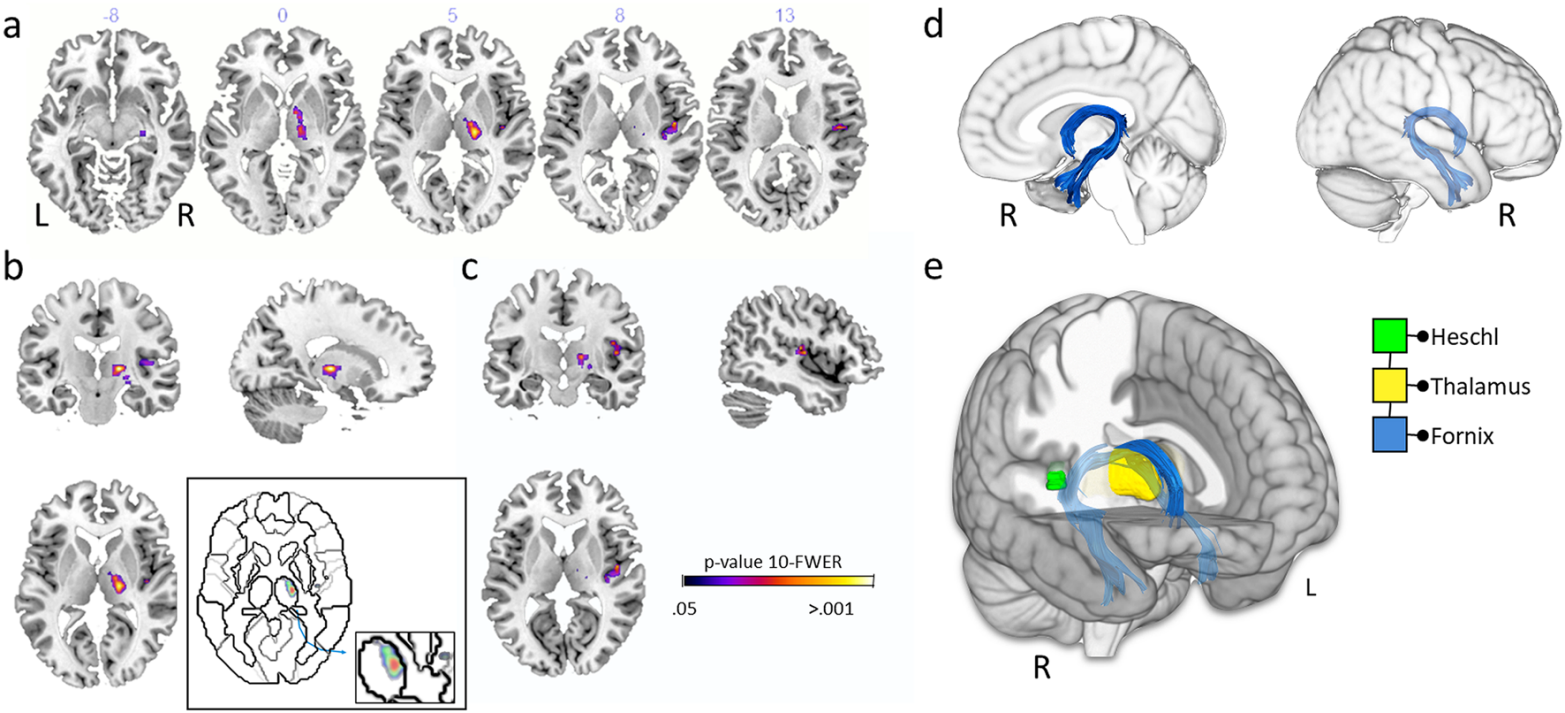
Grey and white matter structures damaged in personal neglect. **a** The lesions from the voxel lesion symptom mapping (Lesymap) and the comparison with the null model (i.e. only clinical variables). Numbers refer to the *Z* in the MNI coordinates. **b** Thalamus (*X* = 18; *Y* = 17; *Z* = 5); in the square, details of the thalamic cluster of lesion are shown. **c** Lesions in the gyrus of Heschl (*X* = 46; *Y* = 12; *Z* = 8). Colour bar represents the *p* statistics resulting from the lesion analyses. 10-FWER: *p* values are calculated from the tenth highest *t* value and familywise error corrected. **d** Medial and lateral view of the fornix. **e** Antero-medial view of the network of personal neglect as resulting from the comparisons of the regression models and including the gyrus of Heschl (green), thalamus (yellow), and fornix (blue). *L* left, *R* right

The linear regression models, however, showed that among all of these grey and white matter structures, only the thalamus (*F*(1,97) = 4.513, *p* = 0.036, η_*p*_^2^ = 0.05), Heschl gyrus (*F*(1,97) = 4.788, *p* = 0.031, η_*p*_^2^ = 0.05) and fornix (*F*(1,97) = 4.247, *p* = 0.042, η_*p*_^2^ = 0.04) could be attributable to the personal neglect symptoms. None of the other tracts and lesions were statistically significant after controlling for extraneous clinical and neuropsychological deficits (all *p* > 0.07, η_*p*_^2^ < 0.02) (Fig. 1).

Finally, the model that takes into account these three structures explains the symptoms better than clinical variables alone (*F*(3, 98) = 3.087, *p* = 0.031, η_*p*_^2^ = 0.09).

## Discussion

The main result of the study is that we were able to identify a brain network associated with PN. It involves cortical and subcortical structures principally sited on the medial side of the right hemisphere and at least two of the elements of the study represent novelties. Firstly, to the best of our knowledge, our sample of patients is currently the largest of any used for the neuroanatomical investigation of PN correlates. Secondly, this is the first study on PN that investigates the white matter via a tractography procedure (although indirect) and does not simply rely on lesion mapping processes that focus on discrete cortical lesions and modular accounts that fail to explain the neural correlates of complex syndromes (Bartolomeo et al. 2007; Doricchi et al. 2008; Pacella et al. 2019; Monai et al. 2020; Moro et al. 2022). Furthermore, the neuroanatomical analysis controls for the impact of clinical variables other than PN.

To date, few group studies have investigated the neuroanatomical correlates of PN, mainly because this condition has been considered as a symptom of a more general spatial neglect syndrome and has been investigated in the literature primarily with reference to extrapersonal space (Committeri et al. 2018 and Caggiano and Jehkonen 2018 for review). The sample of PN patients in these previous studies ranges from 5 to 30 (Bisiach et al. 1986a; Azouvi et al. 2002; Buxbaum et al. 2004; Committeri et al. 2007; Baas et al. 2011; Rousseaux et al. 2015; Caggiano et al. 2020) and the impact of other clinical variables has not been directly investigated. This makes it difficult to disambiguate the lesions that specifically impact on PN and those that are associated with other co-occurrent symptoms. In the present study, a large group of 104 patients was examined, all affected by right hemisphere damage. The investigation used a correlational approach with reference to anatomical data and behavioural scores which allowed us to identify a network specifically involved in PN.

### A network for personal neglect

The first step of the analyses of the grey matter (multivariate VLSM – LESYMAP) revealed a group of cortical areas that had previously been identified as contributing to PN symptoms, in particular, the superior temporal and Heschl’s gyri (Committeri et al. 2007; Baas et al. 2011; Rousseaux et al. 2015) Rolandic operculum (Bisiach et al. 1986a; Azouvi et al. 2002; Buxbaum et al. 2004; Rousseaux et al. 2015; Caggiano et al. 2020), insula (Buxbaum et al. 2004; Caggiano et al. 2020) and hippocampus (Rousseaux et al. 2015). Subcortical structures such as the pallidum (Bisiach et al. 1986a, b; Buxbaum et al. 2004) and thalamus (Bisiach et al. 1986a; Buxbaum et al. 2004) were also involved.

However, when we controlled for concurrent clinical variables (linear regression models with each patient’s lesion size, interval, scores at the MRC, Bisiach test for AHP and line bisection), only Heschl’s gyrus and the thalamus were statistically significant. This suggests that lesions in these two structures account for the symptoms, while the other structures identified in the first step are probably associated with other co-occurrent clinical, neuropsychological variables.

A descriptive analysis of the cluster of lesions in the thalamus (Fig. 1b) revealed that the area which is maximally damaged is the ventral lateral portion of the structure, involving the ventral antero-lateral nucleus, the ventral postero-lateral nucleus and part of the pulvinar (Macchi and Jones 1997). The ventral antero-lateral nucleus is involved in motor control, receiving inputs from the pallidum and brainstem nucleus (i.e. reticular formation, substantia nigra) and sending outputs to the primary and secondary motor cortex. The ventral postero-lateral nucleus contributes to sensory functions, with its inputs coming from the spinal cord and its outputs going towards the primary and secondary somatosensory cortices. Finally, the pulvinar receives inputs from genicular bodies, superior colliculi and the amygdala and spreads its outputs over wide areas in the cortex in the lateral (parietotemporal, prefrontal and orbitofrontal cortices) and medial (cingular cortex) associative cortices (Arcaro et al. 2015). Taken as a whole, these disconnections suggest the possibility that PN is not correlated with only the sensorimotor systems but also involves the integration of cognitive and emotional components.

The finding of a role played by Heschl’s gyrus represents a novelty in this study, although previous studies have had controversial results regarding the superior temporal gyrus, identified sometimes as being more involved in extrapersonal neglect (Committeri et al. 2007) and in other studies pertaining to personal neglect (Baas et al. 2011; Rousseaux et al. 2015). Furthermore, according to some previous findings, PN may be considered as a disorder of body structural representations (i.e. a visuo-spatial, topological map of the body), with deficits that are indeed associated with damage in the superior temporal gyrus (Di Vita et al. 2017, 2019; Boccia et al. 2020). Indeed, the hypothesis of a role of the superior temporal cortex in personal (and extrapersonal) neglect seems to be plausible, as the superior temporal gyrus is a crucial node of the ventral circuit responsible for the reorientation of an individual’s attention in space (Corbetta et al. 2005).

In the analysis of the white matter, the only tract that was significantly associated with PN was the fornix, which is part of the limbic system. This is located in the medial area of the cerebral hemispheres under the corpus callosum, and it connects the hippocampus to the mammillary bodies, the anterior thalamic nuclei and the hypothalamus. In the rostral section, the fornix bifurcates into two columns: (1) the septo-hyppocampal pathway which projects from the hippocampus to the medial septum and nucleus accumbens (Catani and Thiebaut de Schotten 2012) and (2) the subiculo-thalamic pathway which projects from the subiculum to the anterior nuclei of the thalamus and mammillary bodies (Senova et al. 2020). Thus, the fornix represents a main connection between the structures of the medial temporal cortex and the limbic system, playing an important role in emotions and episodic memory. However, its connection pathways, in particular the subiculo-thalamic one, also contribute to the integration of spatial and mnemonic information (Vertes and Kocsis 1997). Unfortunately, in this study we did not systematically collect data on memory but the link between episodic, self-referred memory and personal neglect represents a very interesting topic for further studies.

In contrast with previous studies, in the patients in our sample, the parietal cortex and the temporo-parietal junction were not directly damaged. This was unexpected as various previous studies had found these associative areas, in particular the supramarginal gyrus, to be involved in personal neglect (Bisiach et al. 1986a; Committeri et al. 2007; Baas et al. 2011; Rousseaux et al. 2015). We cannot exclude the possibility that this depends on different recruitment criteria or on the multivariate approach to lesion analysis and the assessment of the lesion size that our analysis of grey matter applied even before checking for other clinical variables. However, our results suggest the possibility that the parietal cortex is disconnected from subcortical structures (i.e. the pulvinar).

Indeed, the study suggests that PN results from the functional integration of combined white and grey matter pathology with both topological and hodological dysfunctions that disrupt the function as a whole (Catani and Ffitche 2005). In other words, it appears likely that the accurate representation of contralesional personal space does not rely on the engagement of a specific module, but rather the integration of sensorimotor and spatial information, potentially also learned expectations about such information processed by the identified functional network of spatially distributed areas and their connections (Catani and Ffitche 2005).

For the first time, the data collected provide evidence that the PN network does not involve only sensorimotor and spatial information (Committeri et al. 2018), but also requires a contribution from deep structures relating to the process of self-body referred information (limbic system). For this reason, PN cannot be simply considered as a disorder affecting an individual’s attention towards or representation of their body space, but rather represents a symptom involving an alteration of the bodily self. Crucially, the PN network does not overlap with the network associated with other body representation disorders, such as a disturbed sensation of body ownership (i.e. asomatognosia and somatoparaphrenia) which involves a fronto-insular–parietal network (Moro et al. 2022), or anosognosia for hemiplegia, which involves the premotor loop, the ventral attentional system and the limbic system (Pacella et al. 2019). Thus, the sense of bodily self is probably widespread across complex multiple networks. How this activity integrates in order to generate the individual’s consistent, continuous experience of unity will be the object of future investigations.

### Diagnosing personal neglect

Although this study is mainly focused on the anatomical correlates of PN, there are results which are also potentially interesting in the clinical field. In some recent reviews (e.g.Committeri et al. 2018; Caggiano and Jehkonen 2018), the use of multiple tests is advised to overcome the limitations inherent in each individual test. In particular, there is evidence that the face and body may be separately impaired and therefore both should be investigated (Committeri et al. 2018). In this study, two tasks were administered to the majority of the participants (the One-Item test was not administered to 18 of the 104 patients recruited). These tests focused on the head and the contralesional hand, respectively. It was found that the sensitivity of the two tests was significantly different, as with the Comb subtest of the Comb and Razor test, 68 out of our 104 patients (65.38%) had results under cutoff (i.e. scores < − 0.011 indicate of PN; McIntosh et al. 2000), while with the One Item test (i.e. scores ≥ 1 indicate PN; Bisiach et al. 1986a), only 31 out of the 86 tested (36.04%) had pathological scores. The consistency between the two tasks was 77.42% when the One Item test was taken into consideration (i.e. PN was consistently diagnosed in 24/31 cases in both tests). However, it would appear that this task underestimates the symptoms of PN (37 patients were diagnosed with PN in the Comb test but were not diagnosed in the One Item test).

Although very easy and quick to administer even in acute post-stroke phases, the One Item test has in reality some limitations, as there is, on one side, the possibility of getting false negative scores (e.g. because the instructions of the test explicitly force the participants to pay attention to and look at the neglected side of their body) and, on the other side, the risk of false positive scores (e.g. a failure to point to the left hand might reflect directional hypokinesia, Heilman et al. 1993). The Comb and Razor test shows some limitations as well, in particular as a result of the fact that it only focuses on the subject’s head instead of on the whole body (as for example in the Fluff Test, see Cocchini and Beschin 2020 for a recent revision of the administration and scoring criteria). Nevertheless, the decision to use the Comb test for this neuroanatomical study was due not only to the availability of data for all of the 104 participants, but also to the fact that there are data from the validation of this tool (McIntosh et al. 2000) with a cutoff which is useful for diagnosis, while the One-item test does not have a validation.

## Limitations and conclusions

A limitation of the study concerns the indirect nature of our functional inferences which are based on structural brain damage and information regarding probable white matter disconnections rather than functional neuroimaging. Unfortunately, the clinical conditions of the patients in our study made it extremely difficult to perform functional neuroimaging due to the size of the sample. Another limitation regards the sensitivity level of the neuroimaging techniques we employed since they do not depict the full extent of the damage produced by stroke lesions (Hillis et al. 2000). However, these limitations mainly apply to small sample studies, while in this study the large number of patients investigated, the procedure used for lesion delineation, the diagnostic criteria and the check carried out for neuropsychological variables reduce these risks. Finally, only right brain-damaged patients were recruited for the study, although the literature on the topic shows that PN may also present with left hemisphere lesions. Further studies are needed to investigate potential differences in symptoms relating to which side of the brain is lesioned.

In conclusion, the study shows that when a large sample of patients is considered, and the method of evaluation is reliable, PN is the result of lesions and disconnections in a network involving the temporal superior cortex, thalamus and its disconnection from the limbic system (via fornix).

## Supplementary Information

Below is the link to the electronic supplementary material.

Supplementary file1 (DOCX 22 KB)

## Data Availability

The datasets analysed during the current study are available in the Open Science Framework repository, https://osf.io/znaq7/?view_only=148d9fc4d077474983d994b992403685.
