# Supplementary material for: Disconnections in personal neglect
Source: Brain Struct Funct. 2022 Jun 7;227(9):3161–71. doi: 10.1007/s00429-022-02511-z (PMC9653363; doi:10.1007/s00429-022-02511-z)
Supplement: Supplementary file 1 — Supplementary file1 (DOCX 22 KB) [file 429_2022_2511_MOESM1_ESM.docx]

# Supplementary Materials

## Disconnections in personal neglect

**S. Bertagnoli, V. Pacella, E. Rossato, P.M. Jenkinson, A. Fotopoulou, M. Scandola & V. Moro**

Correspondence to: Valentina Moro

**NPSY.Lab-VR, Department of Human Sciences, University of Verona, Lungadige Porta Vittoria, 17, 37129 Verona, Italy**

**e-mail: valentina.moro@univr.it**

**Table SM1 Grey matter lesions**

The table shows the number(N>0) and percentage of voxels (%N>0) relating to damaged tissue in each area as resulting in the Voxel Symptoms Lesion Multivariate Mapping (LESYMAP) to find the lesions that correlate with the behavioural score at Comb subtest.

| **Area** | **N>0** | **%N>0** |
| --- | --- | --- |
| Rolandic Operculum Right | 142 | 1.3 |
| Insula Right | 235 | 1.7 |
| Hippocampus Right | 20 | 2.6 |
| Pallidum Right | 24 | 1.1 |
| Thalamus Right | 1117 | 13.3 |
| Heschl Right | 353 | 18.2 |
| Temporal Superior Right | 23 | 0.1 |

**Table SM2 White matter disconnections**

Details of the 34 tracts that result to be disconnected in at least 20% of patients, with means and standard deviations of the probability of disconnection (in %) as resulting from Tractotron.

| **Tract** | **Mean** | **SD** |
| --- | --- | --- |
| Anterior Commissure | 69.1 | 36.1 |
| Anterior Thalamic Projections Right | 95.5 | 15.5 |
| Arcuate Anterior Segment Right | 86.6 | 30.2 |
| Arcuate Long Segment Right | 82.2 | 24.5 |
| Arcuate Posterior Segment Right | 76.0 | 36.8 |
| Cingulum Right Anterior | 60.2 | 42.4 |
| Cingulum Right | 63.6 | 41.0 |
| Cingulum Right Posterior | 36.3 | 42.5 |
| Corpus callosum | 98.3 | 5.8 |
| Cortico Spinal Right | 96.9 | 15.4 |
| Face U tract Right | 51.3 | 38.9 |
| Fornix | 55.6 | 32.6 |
| Frontal Aslant tract Right | 86.5 | 29.5 |
| Frontal Commissural | 81.2 | 34.4 |
| Frontal Inferior longitudinal Right | 74.0 | 37.7 |
| Frontal Orbito Polar Right | 46.6 | 45.0 |
| Frontal Superior Longitudinal Right | 50.6 | 46.7 |
| Fronto Insular tract2 Right | 57.3 | 35.1 |
| Fronto Insular tract3 Right | 83.3 | 31.1 |
| Fronto Insular tract4 Right | 88.9 | 29.1 |
| Fronto Insular tract5 Right | 91.5 | 23.7 |
| Fronto Marginal tract right | 32.1 | 41.4 |
| Fronto Striatal Right | 98.5 | 10.0 |
| Handinf U tract Right | 59.3 | 44.6 |
| Handmid U tract Right | 42.1 | 44.6 |
| Handsup U tract Right | 39.2 | 44.5 |
| Inferior Fronto Occipital fasciculus Right | 92.8 | 22.8 |
| Inferior Longitudinal Right | 81.0 | 35.8 |
| Optic Radiations Right | 67.4 | 34.5 |
| Pons Right | 96.7 | 15.9 |
| Superior Londgitudinal Fasciculus III Right | 94.5 | 17.3 |
| Superior Londgitudinal Fasciculus II Right | 85.0 | 31.3 |
| Superior Londgitudinal Fasciculus I Right | 66.8 | 42.1 |
| Uncinate Right | 72.8 | 41.9 |
